# Study of the Gastroprotective Effect of Extracts and Semipurified Fractions of *Chresta martii* DC. and Identification of Its Principal Compounds

**DOI:** 10.1155/2015/576495

**Published:** 2015-03-17

**Authors:** E. S. Franco, M. E. B. Mélo, B. J. A. Jatobá, A. L. B. D. Santana, A. A. R. Silva, T. G. Silva, M. S. Nascimento, M. B. S. Maia

**Affiliations:** ^1^Department of Physiology and Pharmacology, Laboratory of Pharmacology of Bioactive Products, Federal University of Pernambuco (UFPE), Recife, PE, Brazil; ^2^Department of Parasitology, Laboratory of Mutagenesis and Research Center Aggeu Magalhães, Fiocruz Recife, PE, Brazil; ^3^Department of Antibiotics, Laboratory of Chemistry of Natural Products, UFPE, Recife, PE, Brazil; ^4^Sobral Laboratory of Pharmacology, Federal University of Ceará (UFC), CE, Brazil; ^5^Department of Antibiotics, Laboratory of Bioassays for Research on Drugs, UFPE, Recife, PE, Brazil

## Abstract

*Chresta martii* (Asteraceae) is a species widely used by the population of the Xingu region of Sergipe, Brazil, in the form of a decoction (aerial parts) for the treatment of gastrointestinal diseases. The study aims to assess the gastroprotective activity of organic extracts and semipurified fractions and identify the principal compounds present in *C. martii* responsible for such activity. The organic extracts (cyclohexane: ECCm, ethyl acetate: EACm, and ethanol: EECm) were obtained from the dried aerial parts (500 g) of *C. martii*. For evaluation of the gastroprotective activity of extracts (50, 100, or 200 mg/kg; p.o.), male *Swiss Webster* mice (25–30 g) were used which had gastric ulcers induced by indomethacin (40 mg/kg, s.c.) or ethanol (0.2 mL/animal; p.o.). Among the extracts evaluated, EACm exhibited significant (*P* < 0.05) gastroprotective activity in the models used. The fractionation of EACm was performed in a silica gel column 60 eluted with the following compounds: [chloroform—F1 yield (10%)], [chloroform/ethyl acetate (1/1)—F2 yield (6%)], [ethyl acetate—F3 yield (8%)], and [ethyl/methanol acetate (1/1)—F4 yield (5%)]. Of the fractions described above, the F1 (25 mg/kg; p.o.) had greater gastroprotective activity (*P* < 0.05) than that displayed by ranitidine (80 mg/kg; p.o.) in the ethanol-induced ulcer model. The refractionation of F1 produced 23 subfractions and from these two yellow amorphous compounds were obtained by recrystallization, Rf: 0.46 and 0.31 (ethyl acetate : chloroform 5 : 5). The compounds isolated were characterized by nuclear magnetic resonance spectroscopy (^1^H-NMR and ^13^C-NMR) and identified as flavones: chrysoeriol (yield: 0.43%) and 3′,4′-dimethoxyluteolin (yield: 0.58%). *Conclusion*. Flavone 3′,4′-dimethoxyluteolin is the principal compound present in the species *C. martii* and is probably responsible for gastroprotective activity observed in this species.

## 1. Introduction

Gastric and duodenal ulcers are associated with the imbalance between the protective and aggressive factors of the gastric mucosa [1]. Among the protective factors are prostaglandins, nitric oxide, mucus, and bicarbonate [2, 3]. However, the aggressive factors which predispose the emergence of gastric ulcers are the following: nonsteroidal anti-inflammatory medications (NSAIDs),* Helicobacter pylori* infections, and the chronic use of alcoholic beverages [4, 5]. It is known that this imbalance can significantly impact control of hydrochloric acid secretion by the stomach, whose production has hormonal and neural influences. Hormonal control (endocrine and paracrine) occurs in a complex manner via at least three different cell types: enterochromaffin-like cells producing histamine, G cells producing gastrin, and D cells secreting somatostatin. Neural control is linked to vagal cholinergic control, which acts on all these cells and parietal cells. Histamine, gastrin, and acetylcholine, the latter released by the vagus, directly and positively influence the production of HCl by parietal cells. Somatostatin acts in reverse, inhibiting the release of histamine and gastrin and, consequently, the production of acid [6].

This pathology is the most common chronic illness among adults, affecting 5 to 10% of the world's population. In children, from four to seven new cases of gastric ulcers are registered per year in the major pediatric centers [7]. In both cases, the treatment is carried out using drugs which act by inhibiting the proton pump, neutralizing the acid secretion (antacids) or blocking the histamine receptors (H2 antihistamines) [8]. Although these medications can produce serious adverse reactions, including hypersensitivity, arrhythmia, and impotence [9], they are commonly used for the treatment of gastric ulcers [5].

From this perspective, investigations into the use of medicinal plants are supported by the richness of the Brazilian flora, and the search for new antiulcerogenic agents, most notably with regard to the identification of their principal compounds, elucidation of structure, and pharmacological activity (safety and efficacy), has been the target of constant research [10].

*Chresta martii* (Asteraceae) is popularly known as* muricica.* In the Xingu region, Sergipe, Brazil, this species is widely used in the form of a decoction of aerial parts (±10 g/L) for the treatment of gastrointestinal diseases [11, 12]. Until now, only two studies, using an experimental model of gastric ulcers, have been published reporting the gastroprotective activity of a hydroalcoholic extract of* C. martii*. One of the studies identified a compound belonging to the class of terpenes (sesquiterpene lactone) [13, 14]. Thus, the present study aimed to investigate the gastroprotective activity of different organic extracts and semipurified fractions of* C. martii* in experimental models of gastric ulcers induced by indomethacin or ethanol in* Swiss Webster* mice and identify the class and chemical structure of the principal compounds coming from the product with the best gastroprotective activity.

## 2. Materials and Methods

### 2.1. Botanical Material

*Chresta martii* (DC.) H. Rob. was collected in the Xingu region located in Sergipe, Brazil, at coordinates ranging from −9.5563 to −9.5548 latitude, 37.940 longitude, and an altitude of 130 meters, in January 2011. Samples of the material collected were identified by Dr. Nádia Roque (Institute of Biology in the Department of Botany at the Federal University of Bahia, Brazil) and plant specimens were deposited at the herbarium HUVA (Sobral, Ceará, Brazil) under number 14602.

### 2.2. Animal Model

Male* Swiss Webster* mice (25–30 g) were kept at 22 ± 2°C under a light/dark cycle of 12/12 h, with water and food* ad libitum*. Before the experiment (24 h), the animals were fed with a 5% glucose solution and water* ad libitum*. All treatments and protocols were carried out according to the “Practical Manual on the Ethical Use and Care of Laboratory Animals” of the Brazilian Society of Laboratory Animal Science (SBCAL). The experiments are in accordance with the Guidelines for the Care and Use of Laboratory Animals. The research was approved and licensed (number 23076.015207/2012-42) by the Commission of Ethics on the Use of Animals (CEUA) of the Federal University of Pernambuco, Brazil.

### 2.3. Preparation of Organic Extracts and Semipurified Fractions

To obtain the organic extracts, aerial parts of* C. martii* were used which had been dried in a convection oven at 50°C and pulverized in a knife mill. Subsequently, the material (500 g) was submitted to extraction by maceration at room temperature using organic solvents of increasing polarity: cyclohexane (ECCm), ethyl acetate (EACm), and 96% ethanol (EECm), at a proportion of approximately 10 g/100 mL of solvent. Each extraction cycle lasted 48 hours, using the residue of the former to perform the next extraction, with three repetitions for each solvent [15]. The extracts were concentrated in a rotary evaporator with reduced pressure at 50°C and 90 RPM and/or lyophilized. After the determination of yield, the extracts were stored in amber bottles and kept at a temperature of −20°C (freezer) until the moment of their use, at which time the material was diluted in saline (0.9% NaCl).

The EACm extract (10 g) was submitted to fractionation, giving rise to four semipurified fractions (FI–F4), using silica gel column chromatography (Vetec 0.063–0.20 mm), eluted with chloroform [Fraction I—F1; yield (10%)], chloroform/ethyl acetate (1 : 1) [Fraction II—F2; yield (6%)], ethyl acetate [Fraction III—F3; yield (8%)], and ethyl acetate/methanol (1 : 1) [Fraction IV—F4; yield (5%)]. The semipurified fractions were concentrated in a rotary evaporator with reduced pressure at 50°C and 90 RPM and stored in amber bottles, at a temperature of −20°C (freezer) until the moment of their use, at which time the material was diluted in a saline solution (0.9% NaCl).

### 2.4. Isolation and Identification of the Principal Compound(s) of Fraction (F1)

The F1 (3 g) underwent refractionation using silica gel column chromatography (Vetec 0.063–0.20 mm), eluted with chloroform/ethyl acetate initially (9/1), with a gradual increase in polarity. This procedure resulted in a total of 23 subfractions arising from the mixing of similar samples as verified by thin-layer chromatography (TLC), silica gel 60 F_254_ (precoated sheets of aluminum, Macherey Nagel), revealed with anisaldehyde/sulfuric acid. The subfractions were dried at room temperature for crystallization of the compounds. Then, the residue was resuspended in acetone for recrystallization [16] and filtered to obtain pure crystals. These crystals were identified by nuclear magnetic resonance (NMR) spectroscopy.

### 2.5. Magnetic Resonance Imaging (MRI)

The spectra of ^1^H- and ^13^C-NMR were recorded using the Varian Unity Plus model, 300 MHz for ^1^H and 75 MHz for ^13^C in the Analytical Center of the Fundamental Chemistry Department, UFPE. The chemical shifts (*δ*) were reported in ppm and the coupling constants were reported in Hertz (Hz). Tetramethylsilane (TMS) was used as an internal reference standard. The crystals were solubilized in DMSO-*d*_6_.

### 2.6. Study of the Gastroprotective Effect of the Organic Extracts and Semipurified Fraction of* C. martii*

#### 2.6.1. Gastric Lesions Induced by Indomethacin

Gastric ulcerations were induced in* Swiss Webster* mice (*n* = 6 animals per group) held under fasting (24 h) by the administration of indomethacin (Sigma) (40 mg/kg; s.c.). The animals were pretreated with extracts (ECCm, EACm, or EECm), each in doses of 50, 100, or 200 mg/kg (p.o.), with a positive control of omeprazole (30 mg/kg; p.o.) or a negative control of saline (5 mL/kg; p.o.), 1 h before the administration of indomethacin. A group containing mice (*n* = 3) without ulcer induction received only saline (5 mL/kg; p.o.) to be used as the default physiological group. The doses of extracts were chosen taking into consideration the prior determination of LD_50_ (results not presented) and by comparison with the dose used by Silva et al. [13, 14] for the same plant species and model of ulcer. The animals were euthanized six hours after the ulcerogenic procedure. Their stomachs were removed and opened along the greater curvature. The ulcer index was evaluated using the quantitative method for gauging the extent of erosion and experimental gastric ulcers as described by Szabo et al. [17]. The percentage of inhibition was calculated in relation to the saline group according to the following formula: %inhibition = UIt/UIs × 100, where UIt and UIs correspond to the ulcer indices of the treated and saline groups, respectively.

#### 2.6.2. Ethanol-Induced Gastropathy

The gastric damage was induced by the administration of 99.9% ethanol (0.2 mL/animal) in* Swiss Webster* mice (*n* = 6 animals per group) kept under fasting (24 h) [18]. The animals were treated an hour before induction with EACm (50, 100 or 200 mg/kg; p.o.) or semipurified fractions F1, F2, F3, and F4 (50 mg/kg; p.o.) or only F1 (12.5, 25 or 50 mg/kg; p.o.). The control groups were treated with ranitidine (80 mg/kg; p.o.) as a positive control or saline (5 mL/kg; p.o.) as a negative control. A group containing mice (*n* = 3) without ulcer induction received saline (5 mL/kg; p.o.) to be used as a physiological parameter group. All the animals were euthanized in a CO_2_ chamber 30 min after the injurious procedure. Their stomachs were removed and opened along the greater curvature, washed in saline, fixed between Petri dishes, and photographed (Sony Cyber-Shot Dsc-h2) at 72 dpi resolution (2816 × 2112 pixels). Hemorrhagic or ulcerative lesions were measured and compared to the total area of each stomach through computerized planimetry using the Image J program (National Institutes of Health, 9000 Rockville Pike, Bethesda, Maryland, USA). The percentage of inhibition was calculated in relation to the saline group according to the following formula: %inhibition = UIt/UIs × 100, where UIt and UIs correspond to the indices of ulcers in the treated and saline groups, respectively [18].

### 2.7. Statistical Analysis

All values were expressed as mean ± SD. For the ulcerative/hemorrhagic area, ANOVA followed by the* Bonferroni* test for multiple comparisons was used. The differences were considered significant when *P* < 0.05.

## 3. Results and Discussion

**Figure 1:**
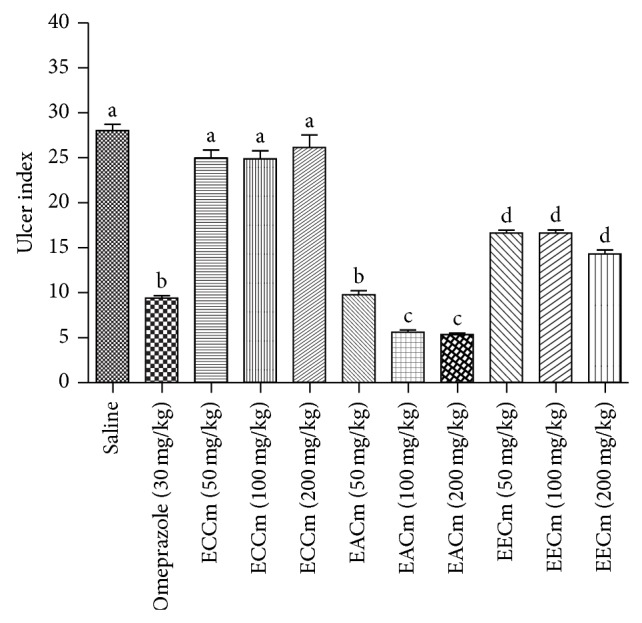
Gastroprotective effect of cyclohexane extract of* C. martii* (ECCm); ethyl acetate extract of* C. martii* (EACm); and ethanolic extract of* C. martii* (EECm) at doses of (50, 100, or 200 mg/kg; v.o.) on gastric lesions induced by indomethacin in mice (*n* = 6). Different letters indicate minimal significance (*P* < 0.05),* Bonferroni* test.

The gastroprotective effect of organic extracts (ECCm, EACm, and EECm) on gastric lesions induced by indomethacin is as shown in Figure 1.

As can be observed, the EACm was the extract which conferred the best gastroprotective activity. The activity observed in the group treated with EACm (50 mg/kg) was significantly (*P* < 0.05) different from the control group (saline) and comparable (*P* > 0.05) to that observed in the group treated with omeprazole (30 mg/kg; p.o.). A previous study using a hydroalcoholic extract of aerial parts of* C. martii* (100 or 400 mg/kg; p.o.) showed significant gastroprotective activity in an ulcer model induced by indomethacin, with inhibition indices of 54.66 and 81.30, respectively [14]. Our results demonstrate superior gastroprotective activity with EACm (100 or 200 mg/kg; p.o.) (79.89% and 80.83%, resp.) taking into consideration the fact that the second dose of EACm was 50% less than the highest dose used by Silva et al. [14]. The best performance observed in our work was due to the extractive process and choice of solvent. These two factors favored the best extraction of substances present in* C. martii*, resulting in significant gastroprotective activity. The ability of indomethacin to induce ulcers experimentally and in humans has been well documented in the literature [19], because it is a potent inhibitor of cyclooxygenases, preventing the biosynthesis of prostaglandins [20]. The reduction in biosynthesis of prostanoids would result in decreased resistance of the gastric mucosa to the action of HCl and pepsin as it induces an imbalance between the protective and aggressive factors of the mucosa [1], taking into account the previous knowledge of the mechanism of action of indomethacin on the gastric mucosa. The likely mechanism of action assigned to EACm can be deduced to involve the biosynthesis of prostaglandins or mimetic action, the same as that which occurs with synthetic analogues of PGE1 (e.g., misoprostol). This mimetic effect can act through stimulation of secretion of mucus and bicarbonate and/or increased blood flow to the stomach.

**Figure 2:**
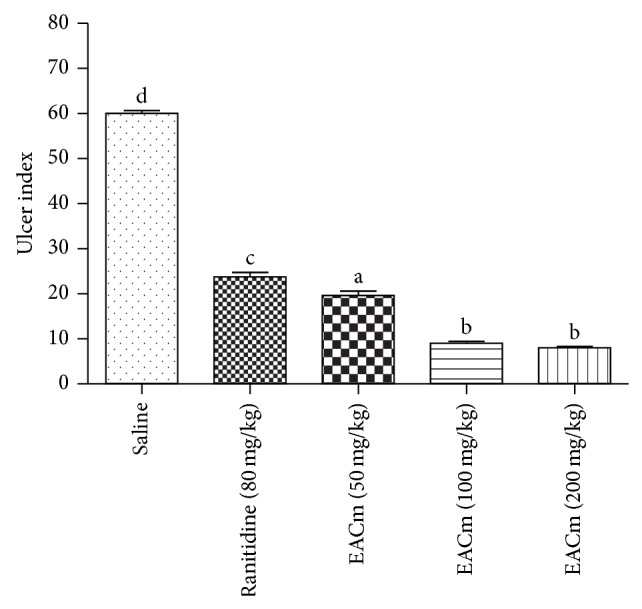
Gastroprotective effect of an ethyl acetate extract of* C. martii* (EACm) (50, 100, or 200 mg/kg; v.o.) in ethanol-induced gastric lesions in mice (*n* = 6). Different letters indicate minimal significance (*P* < 0.05),* Bonferroni* test.

Similar to what was verified with the model of gastric injury induced by indomethacin, EACm (50 mg/kg; p.o.) also presented a cytoprotective effect against ethanol-induced gastric lesions. The gastroprotective effect was dose-dependent and, in all doses tested, superior to that verified with ranitidine (Figure 2).

Ethanol-induced gastric lesions involve different mechanisms such as a reduction in bicarbonate secretion, a reduction in mucus production, damage to gastric blood flow, and direct injury to mucosal cells [21, 22]. These lesions are associated with the excessive production of free radicals, which attack essential cellular constituents such as nucleic acids, proteins, and lipids [23]. The increase of the content of lipid peroxides and oxygen-derived free radicals results in significant changes at the cellular level and causes damage to membranes, cell death, exfoliation, and epithelial erosion [21]. Studies have demonstrated the presence of flavonoids in species belonging to the family Asteraceae and this chemical class has been shown to have important antioxidant properties. This could be one of the forms of action of the compounds present in EACm in providing significant cytoprotection, given that free radicals are one of the harmful factors of the gastric mucosa induced by ethanol.

**Figure 3:**
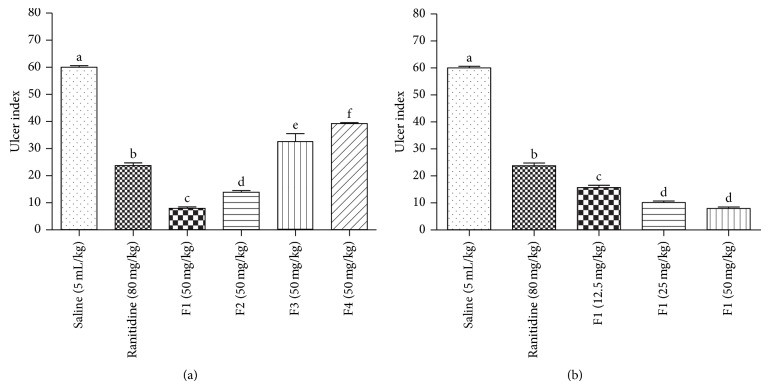
Gastroprotective effect of fractions (F1, F2, F3, and F4) (50 mg/kg; v.o.) (a) and the fraction (F1) in isolation (50, 25, or 12.5 mg/kg; v.o.) (b) obtained from the ethyl acetate extract of* C. martii* on ethanol-induced gastric lesions in mice (*n* = 6). Different letters indicate minimal significance (*P* < 0.05),* Bonferroni* test.

Evaluation of the semipurified fractions (F1, F2, F3, and F4) (50 mg/kg; p.o.) against the ethanol-induced ulcer model revealed that animals treated with F1 and F2 showed lower rates of ulcers when compared to the ranitidine control. However, when compared with each other, F1 presented a significantly lower ulcer index (Figure 3(a)). Additionally, the gastroprotective effect presented by F1 was dose-dependent (Figure 3(b)).

**Figure 4:**
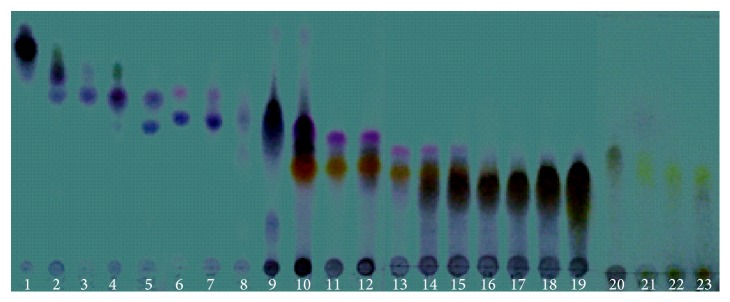
Chromatographic profile by TLC of the eluded fraction (F1) (chloroform : ethyl acetate 8 : 2), revealed with anisaldehyde/sulfuric acid.

After verification of the significant gastroprotective activity of F1, it was refractioned, giving rise to 23 subfractions in which the presence of bands was determined through TLC, revealing anisaldehyde/sulfuric acid, characteristics of flavonoids (yellow), steroids (lilac), and terpenoids (blue) (Figure 4). The existence of a larger number of bands with colors ranging from orange to yellow can be seen. Two pure substances F1.1 (Rf: 0.31) and F1.2 (Rf: 0.48) were recrystallized, showing a yellow, amorphous physical appearance.

The NMR (^1^H and ^13^C) presented peaks from which it was possible to calculate the chemical shift (*δ*) in ppm and the coupling constants in Hertz (Table 1).

**Figure 5:**
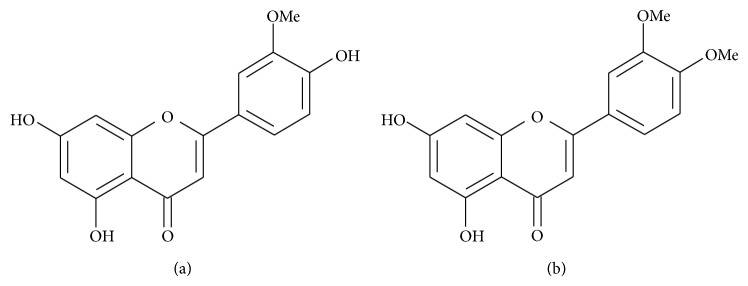
Chemical structure of chrysoeriol (a) and 3′,4′-dimethoxyluteolin (b) isolated from* Chresta martii*.

From the interpretation of the spectra, two flavones were identified: chrysoeriol (F1.1) and 3′,4′-dimethoxyluteolin (F1.2) (Figures 5(a) and 5(b)). The yield of 3′,4′-dimethoxyluteolin was 0.58% and was higher than that observed for chrysoeriol at 0.43%. It was verified in this study that the principal compounds present in EACm are flavones.

This study is a pioneer in the identification of two flavones of* Chresta martii*. According to Van and Lea [24], flavones present at least four important physiological mechanisms: they (1) bind to enzymes in the cell membrane, (2) bind to heavy metal ions, (3) participate in electron transfer of enzymatic systems, and (4) sequester free radicals.

The flavone (3′,4′-dimethoxyluteolin), among other flavonoids, was assessed as to its gastroprotective and anti-inflammatory potential (in different experimental models of gastric ulcers and inflammation using rodents). The mechanism of gastroprotective action proposed for these compounds has been reported to be the metabolic activation of arachidonic acid via cyclooxygenase activation leading to biosynthesis of prostaglandins, such as PGE2 and PGI2, both with important protective function of the gastric mucosa. The compounds were furthermore able to inhibit the activation of 5-lipoxygenase (leukotrienes), which is an important inflammatory mediator, besides acting in the sequestration of free radicals. These results have given rise to some patents by Yoo et al. [25] (EPO 004541), Yoo et al. [26] (US 6025387), and Yoo et al. [27] (EPO 0915864B1). Similarly, Sadik et al. [28] verified potent antioxidant and inhibitory activity of lipoxygenase in a culture of rabbit reticulocytes when different flavonoids were used including luteolin, whose structure is very similar to 3′,4′-dimethoxyluteolin.

However, the fraction (F1) is a mixture (terpenes, flavonoids, and steroids) and these compounds can act synergistically with regard to the gastroprotective potential presented here, possibly involving other mechanisms of action beyond those cited by Yoo et al. [25–27], such as activation of alpha-2 adrenergic receptors as proposed by Silva et al. [13] when evaluating the hydroalcoholic extract of* C. martii* in an ethanol-induced ulcer model.

There is a scarcity of studies that assess the phytochemical profile, at the level of identifying the principal compounds, for* C. martii*. Such work was pioneered by Silva et al. [13, 14] in identifying two sesquiterpene lactones directly from a hydroalcoholic extract. The present study documents for the first time identification of two flavones (3′,4′-dimethoxyluteolin and chrysoeriol) from the genus* Chresta*, with relevant evidence for them being the agents responsible for the gastroprotective effect seen in* C. martii*, given that this investigation occurred through direct monitoring between gastroprotective pharmacological activity and processes of extraction, purification, and identification of compounds. Finally, the results presented here provide rational support for the purported use of the species in the treatment of gastrointestinal disorders, according to ethnopharmacological information.

## Figures and Tables

**Table 1:** Data of  ^1^H- and ^13^C-NMR of flavonoids (F1.1 and F1.2) isolated from the F1 of *C. martii* (300 MHz) in DMSO-*d*_6_.

	Flavonoid (F1.1)		Flavonoid (F1.2)	
Positions	Chrysoeriol		3′,4′-Dimethoxyluteolin	
	*δ* ^1^H (*J*, Hz)	*δ* ^13^C	*δ* ^1^H (*J*, Hz)	*δ* ^13^C
2		163.7		163.9
3	6.88 (*s*)	104.1	6.97 (*s*)	106.0
4		181.8		181.5
5		162.0		163.0
6	6.19 (*d.* 1.8)	98.0	6.21 (*d*. 2.1)	98.2
7		164.2		164.7
8	6.52 (*d. *1.8)	94.0	6.53 (*d*. 1.8)	94.0
9		157.4		157.9
10		104.0		104.0
1′		121.0		120.6
2′	7.56 (*d. *2.4)	110.0	7.56 (*d*. 2.1)	109.8
3′		148.1		148.0
4′		150.8		151.7
5′	6.94 (*dd.* 8.4; 2.4)	116.0	7.13 (*d*. 8.4)	116.2
6′	7.55 (*dd.* 8.4; 2.4)	122.0	7.68 (*dd*. 9.6; 1.8)	121.6
5-OH	12.97			
8-OH	10.79			
4′-OH	9.96			
3′-OCH_3_	3.89 (*s*)	56.0	3.85 (*s*)	56.0
4′-OCH_3_			3.88 (*s*)	56.0
